# Prevalence and severity of syndrome Z in women with metabolic syndrome on waiting list for bariatric surgery: a cross-sectional study

**DOI:** 10.1186/s13098-017-0269-2

**Published:** 2017-09-20

**Authors:** Eduardo Araujo Perez, Luis Vicente Franco Oliveira, Wilson Rodrigues Freitas, Carlos Alberto Malheiros, Elias Jirjoss Ilias, Anderson Soares Silva, Jessica Julioti Urbano, Patricia Clemente Oliveira, Felipe X. Cepeda, Luciana M. M. Sampaio, Ivani C. Trombetta, Humberto Delle, Daniel Gianella Neto, Sergio Roberto Nacif, Roberto Stirbulov

**Affiliations:** 1 Santa Casa School of Medicine, Sao Paulo, SP Brazil; 2 Sleep Laboratory, Nove de Julho University (UNINOVE), Sao Paulo, SP Brazil; 3 University Center of Anapolis (UniEVANGELICA), Anapolis, GO Brazil; 4 Rua Itapicuru 380, Apto 111, Perdizes, Sao Paulo, SP CEP 05006-000 Brazil; 5 Heart Institute (InCor), Hospital das Clinicas da Faculdade de Medicina da, Universidade de Sao Paulo, Sao Paulo, SP Brazil; 6 Nove de Julho University (UNINOVE), Sao Paulo, SP Brazil; 7 Hospital Servidor Público Estadual (HSPE), Sao Paulo, SP Brazil

**Keywords:** Metabolic syndrome X, Obstructive sleep apnoea, Severe obesity, Polysomnography

## Abstract

**Background:**

In recent years, obesity has become one of the most important public health problems in the world, with a growing prevalence in both developed and developing countries. Recent studies show that sleep disturbances, especially obstructive sleep apnoea (OSA) may be a manifestation of metabolic syndrome (MetS). Although the association of OSA with the MetS is largely attributed to obesity, the exact pathophysiological mechanisms and their individual characteristics still need to be identified. This study investigated the prevalence and severity of syndrome Z in obese women with MetS on waiting list for bariatric surgery.

**Methods:**

In this double-center cross-sectional study, female patients aged ≥18 years, stage III severe obesity with MetS, on waiting list for bariatric surgery were recruited. The diagnosis for MetS was made according to the criteria of the national cholesterol education program, adult treatment panel III. Clinical, anthropometric, demographic, biochemistry, and sleep measurements were collected. Correlations between continuous variables with sleep parameters were performed using the Pearson correlation test or Spearman correlation test.

**Results:**

The mean age of 83 patients was 44.8 ± 11.2 years and mean BMI was 42.6 ± 8.1 kg/m^2^. There was a significant correlation between OSA and metabolic score (r = 0.336; P = 0.002), neck circumference (r = 0.218; P = 0.048), basal systolic blood pressure (r = 0.280; P = 0.01), total cholesterol (r = 0.277; P = 0.011) and abdomen circumference (r = 0.284; P = 0.009). The mean values of excessive daytime sleepiness were 10.5 ± 7 demonstrating a value considered normal for its presence. However, a high risk for OSA was observed in practically the entire population. It was observed that the prevalence of Syndrome Z (75.9%) increased significantly according to apnoea hypopnoea index (AHI) (P for trend <0.0000). A prevalence of 27.71% for mild OSA, 20.48% for moderate OSA, and 27.71% for severe OSA was observed. An association of AHI severity with all components of MetS was also observed.

**Conclusions:**

We can conclude that syndrome Z presents a high prevalence in a female population with MetS and a considerable severity according to the presence of OSA. Therefore, patients with MetS should be investigated for the presence of sleep disorders.

*Trial registration* The study has been registered on ClinicalTrials.gov NCT02409160 and followed the standards of The Strengthening the Reporting of Observational Studies in Epidemiology (STROBE) Statement: guidelines for reporting observational studies

## Background

In recent years, obesity has become one of the most important public health problems in the world, with a growing prevalence in both developed and developing countries. This epidemic can be attributed to the modern lifestyle characterized by lack of physical activity and consumption of diets rich in fat [1, 2].

The obesity epidemic results in important health consequences for different ethnic populations. The increase in the number of obese people is associated with increases in the prevalence of comorbidities such as type 2 diabetes, hyperlipidemia, hypertension, obstructive sleep apnea (OSA), heart disease, stroke, asthma, weight bearing degenerative problems in the lower back and extremities, cancer and depression [3–5]. The importance of obesity in the development of cardiometabolic disease is highlighted every day [6].

Since metabolic syndrome (MetS) is strongly linked to obesity, the continuing rise in the prevalence of obesity worldwide is closely followed by increased rates of MetS. The national cholesterol education program (NCEP) adult treatment panel III (ATP III) introduced the term MetS in 2001, as a risk partner to elevated low-density lipoprotein (LDL) cholesterol in cholesterol guidelines [7, 8].

The term MetS is defined by a constellation of risk factors that usually accompany obesity and are associated with a higher risk for atherosclerotic cardiovascular disease (CVD) and type 2 diabetes [9, 10].

The MetS is a disorder characterized by abdominal obesity, arterial hypertension, increased blood triglycerides, decreased high-density lipoprotein (HDL) cholesterol and increased blood glucose [11, 12]. Accumulating evidence indicates that insulin resistance and an increased amount of abdominal fat may be the pathogenic factors responsible for the variety of symptoms of the metabolic syndrome [13–15].

Recent studies show that sleep disturbances, especially OSA may be a manifestation of MetS [16, 17]. These observational studies have shown that OSA and MetS exhibit similar pathophysiological substrates for CVD, where increased blood pressure is a common consequence of these two pathologies. This raises another discussion whether MetS and OSA may have an additive effect on cardiovascular risk factors.

OSA is characterized by recurrent partial or complete collapse of the upper airway in the presence of ventilatory effort during sleep [18]. These events are often associated with recurrent nocturnal oxyhemoglobin desaturation, fragmented sleep, major fluctuations in blood pressure, and increased sympathetic nervous system activity and micro-arousal during sleep [19].

Several risk factors have been identified that contribute to the presence of respiratory sleep disorders (RSD) which include anatomical, mechanical and tissue changes of the upper airway, altered neuromuscular function and instability of the ventilatory control during sleep, and these factors predominate in individual cases, generating different “phenotypes” of OSA [20].

The main risk factors associated with OSA are age, male gender, body mass index (BMI), neck circumference, and craniofacial changes [21]. There is a clear relationship between OSA and cardiovascular risk, neuropsychological problems, reduction in quality of life and consequent increased use of health resources [22–24].

On the prevalence of OSA, three large cohort studies conducted in the United States of America: the Wisconsin sleep cohort study [25], the sleep heart health study [26] and the Penn State cohort [27] observed the presence of estimated sleep disturbances between 6.5 and 9% in women and between 17 and 31% in men according to an apnea–hypopnea index (AHI) of more than five events per hour [25, 27].

A recent survey conducted with a representative population of the city of Sao Paulo showed that 24.8% of men and 9.6% of women presented OSA [28]. A recent study observed a prevalence of about 34% in men aged 30–70 years and 17% in women aged 30–70 years [29].

The HypnoLaus, a population-based study conducted in Lausanne, Switzerland is the lastest study on the prevalence of sleep-disordered breathing in the general population. The authors invited a cohort of 3043 consecutive participants from the CoLaus/PsyCoLaus study to take part. Polysomnography data from 2121 people were included in the final analysis. The median AHI was 6.9 events per hour (IQR 2. 7–14.1) in women and 14.9 per h (7.2–27.1) in men. The prevalence of moderate-to-severe sleep-disordered breathing (≥15 events per h) was 23.4% (95% CI 20.9–26.0) in women and 49.7% (46.6–52.8) in men [30].

There is already considerable scientific evidence based on human and animal studies suggesting that OSA can influence all aspects of MetS, including obesity [31], insulin resistance [32], and dyslipidemia [33, 34]. Other studies have also shown the coexistence of these two pathologies [35, 36]. It has been suggested that the metabolic syndrome “syndrome X” may include OSA and must then be called “syndrome Z” [37].

Although the association of OSA with the metabolic syndrome is largely attributed to obesity, the exact pathophysiological mechanisms and their individual characteristics still need to be identified. This study was performed to investigate the prevalence and severity of syndrome Z in obese women with metabolic syndrome on waiting list for bariatric surgery.

## Methods

### Subjects and recruitment

In this double-center cross-sectional study, women patients with metabolic syndrome on the waiting list for bariatric surgery undergoing polysomnography (PSG) in the sleep laboratory of the Nove de Julho university (UNINOVE), Sao Paulo (SP), Brazil, between June 2015 and September 2016 were evaluated for enrollment. These patients were referred for PSG from the surgery unit of the Hospital of Santa Casa de Misericordia and Hypertension Unit of Hospital das Clinicas da Faculdade de Medicina da Universidade de Sao Paulo (HCFMUSP) from the city of Sao Paulo (SP), Brazil. Participants were recruited consecutively, according to the eligibility criteria of the research protocol.

### Eligibility criteria

Were recruited female patients, aged ≥18 years, with stage III severe obesity (BMI of ≥40 or ≥35 kg/m^2^ with comorbidities) on the waiting list for bariatric surgery, diagnosed with MetS according to the criteria of the national cholesterol education program, adult treatment panel III (NCEP ATP III) [8], with a documented history of failed conventional weight loss attempts, and the ability to understand and agree to participate in the study, based on a signed informed consent. The MetS was defined as the presence of three or more of the following criteria: waist circumference >88 cm, fasting glucose ≥100 mg/dl or treatment with oral agents, triglycerides ≥150 mg/dl or treatment for it, HDL cholesterol <50 mg/dl or lipid-lowering treatment, blood pressure ≥130/85 mmHg or taking antihypertensive [7, 8]. The exclusion criteria will be BMI of >55 kg/m^2^, alcohol or drug abuse, cancer, and any other cardiorespiratory and/or neurological disorders.

### Ethics and trial registration

**Fig. 1:**
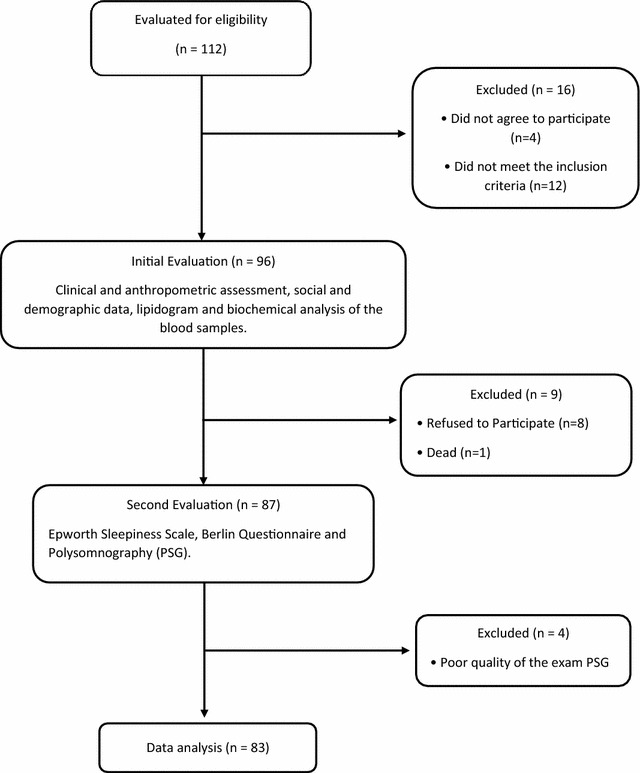
Flowchart of the study

Informed consent was obtained from all patients prior to entry into the study. All patients were given an information sheet detailing the purpose of the study and the study procedure. This study was conducted according the principles of the Declaration of Helsinki, and approved by the human research ethics committees of Nove de Julho university (UNINOVE; Protocol Number 220506/2009) and Irmandade da Santa Casa de Misericordia de Sao Paulo, Brazil (Protocol Number 742.865/2014), and registered at ClinicalTrials.gov (NCT02409160). This protocol followed the STrengthening the Reporting of OBservational studies in Epidemiology (STROBE) [38] and the study has been registered on ClinicalTrials.gov NCT02409160. The Fig. 1 show the flow diagram of the study.

### Clinical, anthropometric and demographic measurements

Clinical, anthropometric and demographic measurements were collected in all included patients: medical history, medication use, blood pressure in sitting position (three measurements), BMI (kg/m^2^) [39, 40], neck circumference (at laryngeal prominence) [41] and waist circumference (measured at the last rib and the iliac crest) [42].

### Laboratory measurements

After fasting overnight, venous blood samples were obtained. Glycose, total cholesterol (TC), HDL cholesterol, LDL cholesterol and triglycerides were measured. Fasting cholesterol, triglyceride (Bayer Corporation, Tarrytown, NY, USA), and HDL cholesterol (Sigma Diagnostics, St. Louis, MO, USA) concentrations were measured using an immune colourimetric assay on an ADVIA^®^ 1650 chemistry system (Bayer Corporation, Tarrytown, NY, USA). LDL cholesterol were derived using the Fried Wald equation. Fasting glucose were measured using a glucose-oxidase-based assay (YSI 2300, Analytical Technologies, Farnborough, UK).

### Sleep assessment

All patients underwent PSG within a maximum period of 15 days after blood collection. Sleep studies were carried out at the Nove de Julho University’s sleep laboratory, by trained technicians using the system with 16-channel PSG Somnologica Studio–Embla A10 version 3.1.2 (Flaga Hs.Medical Devices, Reykjavik, Iceland), recording electroencephalography (EEG) (C4A1, C3A2, O2A1, O1A2), bilateral electrooculography (EOG), submental and bilateral tibialis anterior electromyography (EMG), electrocardiography (EKG), oxygen saturation, body position, thoracic and abdominal movements, oronasal flow by thermistor, nasal flow by cannula and snoring. All exams were read and scored manually blinded to clinical data by certified physicians experienced in sleep medicine according to the criteria of the American Academy of Sleep Medicine (AASM) manual for sleep scoring version-2 which was published in 2012 [18].

Apnoea and hypopnoea were defined according to the criteria as recommended by the AASM. Apnea was defined as the absence or reduction of over 90% of the respiratory signal with duration of at least 10 s. The hypopnea was defined as an airflow reduction between 30 and 90% with a minimum of 10 s accompanied by an oxygen desaturation more than 4% and/or arousals. Were defined sleep fragmentation based on the number of awakenings per hour. Oxygen desaturations were measured as the percentage of recording time with oxygen saturation below 90% (T < 90).

The severity of OSA was defined as AHI >5 events/h. The AHI was calculated by dividing the total number of respiratory events by the total number of hours of sleep. Severity of OSA was graded as, mild OSA: AHI ≥5 and <15 events/h, moderate OSA: AHI ≥15 and <30 events/h, and severe OSA: AHI ≥30 events/h. Patients without OSA were referred to as normal [18].

### Statistical analysis

The data with normal distribution are represented in mean and standard deviation, and the data with abnormal distribution are represented in median and interquartile range. The categorical data are in absolute numbers and the percentage of the total. First, the Kolmogorov–Smirnov normality test was performed. The values of pre- and post- bronchodilator spirometry were compared using the student’s test. Correlations between continuous variables with PSG parameters were performed using the Pearson correlation test or Spearman correlation test. The Statistical Package for Social Sciences SPSS 19.0^®^ (Chicago, IL, USA) was used for the statistical treatment. Statistical significance was set at 5% for all tests (P < 0.05).

## Results

From an initial sample of 142 eligible patients, 112 female patients were recruited during routine medical appointments at the Bariatric Surgery Outpatient Clinic of Santa Casa de Misericordia in Sao Paulo and at the Heart Institute (InCor), University of Sao Paulo’s Clinics Hospital (SP), Brazil with clinical diagnosis of severe obesity with MetS, on the waiting list for bariatric surgery. According to the inclusion and exclusion criteria, some patients were excluded as shown in the study flow diagram (Fig. 1). The final analysis of the data counted on 83 patients.

The clinical, demographic, anthropometric and major comorbidities of the eighty-three patients involved in the study are presented in Table 1. The mean age was 44.8 ± 11.2 years and mean BMI was 42.6 ± 8.1 kg/m^2^. There was no significant difference between the variables age, weight, BMI, tobacco and alcohol consumption showing homogeneity of the sample. However, when comparing waist and neck circumference values, a significant difference was observed when grouped according to the severity of OSA. Regarding ethnicity, we observed the predominance of Caucasoid. Previous treatment, 46 (55.4%) patients taking antihypertensive drugs, 27 (32.5%) patients receiving oral antidiabetic agents and 10 (12%) patients taking hypolipemiant drugs.

**Table 1:** Demographic and anthropometric characteristics of apneic and non-apneic patients

Variables	All patients	No-OSA	Mild OSA	Moderate OSA	Severe OSA	P
	(n = 83)	(n = 20)	(n = 23)	(n = 17)	(n = 23)	
Age	44.8 ± 11.2	49.5 ± 10.5	48.7 ± 12	46.5 ± 10.6	48.8 ± 13.6	
Weight (kg)	108 ± 19	104.9 ± 21.1	108.1 ± 14.9	103.8 ± 16	112.3 ± 22.6	
BMI (kg/cm^2^)	42.6 ± 8.1	40.1 ± 8	41.2 ± 6.6	40.3 ± 6	41.9 ± 8.2	
Waist (cm)	119.4 ± 14.1	111.6 ± 13.1	119.7 ± 11.1	126.7 ± 14.8^a^	129.4 ± 10.8^a, b^	***
Neck (cm)	40.3 ± 3.4	38.9 ± 2.8	40.9 ± 3.6	41.7 ± 3.2^a^	43.3 ± 2.5^a, b^	***
Ethnicity						
Caucasoid (%)	65.3	70	16.8	14.4	14.4	
Negroid (%)	34.6	7.2	10.8	6.0	13.2	

*OSA* obstructive sleep apnea, *BMI* body mass index, *Kg* kilogram, *cm* ^2^ square centimeters, *SD* standard deviation

*** P < 0.001; data are expressed as mean (± SD) and percentage (%)

^a^no-OSA vs moderate OSA or severe OSA

^b^mild OSA vs severe OSA

Table 2 shows the clinical variables related to peripheral blood pressure and blood biochemical analysis of patients stratified according to the presence of OSA or not and according to their severity levels. Significant difference was observed regarding systolic blood pressure (SBP) between the mild, moderate and severe groups in relation to the group of patients who did not present OSA. Regarding glycose levels, a significant difference was observed comparing the moderate and severe groups with the non-OSA group. The other variables not presented statistical significance.

**Table 2:** Clinical and metabolic variables of the apneic and non-apneic patients

Variables	All patients	NO-OSA	Mild OSA	Moderate OSA	Severe OSA	P
	(n = 83)	(n = 20)	(n = 23)	(n = 17)	(n = 23)	
SBP (mmHg)	137.6 ± 11.6	126.2 ± 7.4	136 ± 11.6^a^	139.1 ± 8.3^a^	148.1 ± 5.4^a, b, c^	***
DBP (mmHg)	86.0 ± 5.3	84.5 ± 7	85 ± 3.9	85.9 ± 5.3	88.5 ± 4	
Glycose (mg/dl)	124.3 ± 34.3	99.8 ± 14.5	117.6 ± 22.6	139.7 ± 41.6^a^	142.3 ± 36.5^a^	***
TC (mg/l)	237.2 ± 30.2	183.7 ± 118.1	198.3 ± 36.8	186.2 ± 30.5	181.5 ± 38.3	
Tg (mg/dl)	187.6 ± 64.9	221.9 ± 41.9	244 ± 25.7	234.7 ± 25.5	245.6 ± 20.1	
HDL (mg/dl)	40.1 ± 6.0	42.3 ± 7.5	41.2 ± 7.2	39 ± 6.6	38 ± 4.1	
LDL (mg/dl)	132.5 ± 33.1	140.6 ± 30.6	128 ± 30.3	124.8 ± 31.6	135.5 ± 38.8	
VLDL (mg/dl)	312.5 ± 17	39.2 ± 22.4	26.6 ± 12	28.9 ± 12.8	31.1 ± 17.4	

*OSA* obstructive sleep apnea, *SBP* systolic blood pressure, *DBP* diastolic blood pressure, *TC* total cholesterol, *Tg* triglycerides; *HDL* high density lipoprotein, *LDL* low density lipoprotein, *VLDL* very low density lipoprotein, *SD* standard deviation

*** P < 0.001; data are expressed as mean (± SD)

^a^no-OSA vs mild OSA, moderate OSA or severe OSA

^b^mild OSA vs severe OSA

^c^Moderate OSA vs severe OSA

The PSG variables are shown in Table 3 according to the presence and severity of OSA. In relation to the total sleep time and sleep efficiency, no significant difference was observed. This shows that all patients slept well exceeding 85.5% of total record time. However, in relation to AHI, obstructive apnea index, central apnea index, mixed apnea index and hypopnea indices, a significant difference was observed when the groups were compared by severity. Also, the values referring to oxygen saturation and maximal heart rate (HR) presented significant differences.

**Table 3:** Polysomnographic variables

Variables	NO-OSA	Mild OSA	Moderate OSA	Severe OSA	P
	(n = 20)	(n = 23)	(n = 17)	(n = 23)	
TST (min)	368.3 ± 72.7	348.1 ± 41.1	354.8 ± 36.6	355.5 ± 60.8	
SE (%)	90.7 ± 5.6	89.5 ± 10	87.2 ± 6.9	85.9 ± 8.6	
NREM1%	3.8 ± 1.9	9.6 ± 16	6.1 ± 2.1	7.1 ± 2.5	
NREM2%	56.5 ± 7.2	50.5 ± 11.9	52.6 ± 12	55.3 ± 5.6	
NREM3%	25.5 ± 7.8	25.8 ± 9.6	27.6 ± 8.8	25.7 ± 6.4	
REM%	14.1 ± 7.3	13.6 ± 5.3	11.3 ± 6	11.1 ± 6.9	
AHI/h	3.4 ± 0.9	9.9 ± 3.3	21.2 ± 5.5^a, b^	52 ± 14.5^a, b, c^	***
OAI/h	5.5 ± 4.6	6.6 ± 4.2	30.6 ± 30.4	88 ± 67.2^a, b, c^	***
CAI/h	0.5 ± 0.5	1.7 ± 1.3	1.6 ± 0.8	3.4 ± 3	***
MAI/h	0	1 ± 0	1 ± 0	4.8 ± 4.1	***
Hypop/h	2.4 ± 0.6	8.6 ± 3.3	16.1 ± 5.9	35.4 ± 14.9^a, b, c^	***
SpO2 vigília	95.9 ± 1.6	94.5 ± 1.3	95 ± 1.6^a^	93.2 ± 2.1^a, b, c^	*
SpO_2_ sleep	94.6 ± 2.5	93.6 ± 1.9	93.7 ± 1.2	89.5 ± 4.3	***
SpO_2_ <90% TST (%)	0.4 ± 0.2	23.5 ± 35	22.5 ± 38.4	39.3 ± 35.1	***
SpO_2_ <80% TST (%)	0	8.5 ± 0.3	9.8 ± 3.1	11.3 ± 3.5	***
SpO_2_ <70% TST (%)	0	2.8 ± 1.9	3.5 ± 1.7	4 ± 2.1	***
RH wake	87 ± 7.4	85 ± 8.6	88 ± 10.5	87.5 ± 13.2	
HR med. sleep	81.6 ± 7.5	75.5 ± 14.1	73 ± 8.8	72.5 ± 9.1	
HR máx. sleep	137.6 ± 43.6	125.4 ± 22.6	104.6 ± 15.8^a^	103.2 ± 16.0^a, b^	**
HR min. sleep	48.2 ± 8.5	53.1 ± 14.1	41.1 ± 12.5	46.8 ± 6.8	
ODI/h	4.5 ± 5	6.9 ± 4	17.3 ± 8.1	50.1 ± 24.2	

Data are expressed as mean (± SD) or no (%)

*OSA* obstructive sleep apnea, *TST* total sleep time, *SE* sleep efficiency, *NREM1* sleep stage 1 without rapid eye movements, *NREM2* sleep stage 2 without rapid eye movements, *NREM3* stage 3 of sleep without rapid eye movements, *REM* sleep with rapid eye movement, *AHI* apnea/hypopnea index per hour of sleep, *OAI* obstructive apnea index per hour of sleep, *CAI* central apnea index per hour of sleep, *MAI* mixed apnea index per hour of sleep, *Hipop*/*h* hypopnea index per hour of sleep, *SpO*_2_ peripheral oxyhemoglobin saturation, *HR* heart rate, *ODI* oxyhemoglobin desaturation index, *Min* minutes, *SD* standard deviation

* P < 0.05; ** P < 0.01; *** P < 0.001

^a^No-OSA vs moderate OSA or severe OSA

^b^Mild OSA vs moderate OSA or severe OSA

^c^Moderate OSA vs severe OSA

**Fig. 2:**
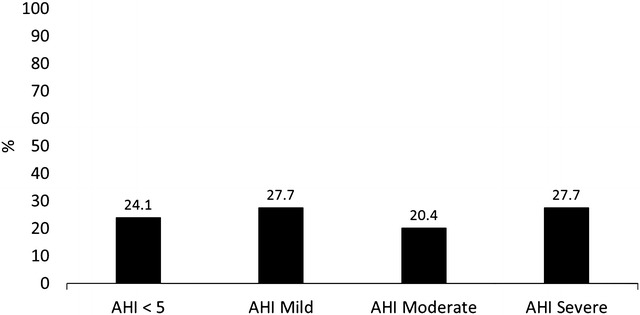
Prevalence and severity of syndrome Z in patients with metabolic syndrome. AHI = apnea/hypopnea index per hour of sleep

Our sample was composed of severe obese women with MetS. In Fig. 2 we can observe that the prevalence of “Z” Syndrome (75.8%) increased significantly according to AHI (P for trend <0.0000). We can also observe that according to AHI criteria, 20 subjects had no OSA (24.1%), 23 had mild OSA (27.7%), 17 had moderate OSA (20.4%), and 23 had severe OSA (27.7%).

**Fig. 3:**
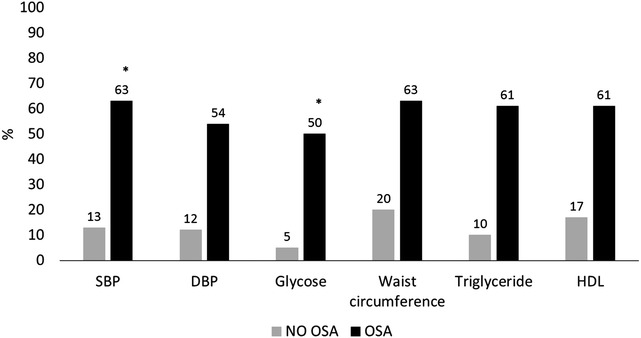
Prevalence of metabolic syndrome components according absence and severity of obstructive sleep apnea. SBP = systolic blood pressure; DBP = diastolic blood pressure; HDL = high density lipoprotein; OSA = obstructive sleep apnea; AHI = apnea–hypopnea index. (*P < 0.05)

Figure 3 shows the prevalence of the each of the components of the MetS according to the absence and severity of OSA. It can be observed a higher prevalence of all components of MetS in patients with OSA, however, only a significant difference was observed regarding the values of SBP and glycose.

Table 4 shows a significant association between some components of MetS (HDL <40/50, fasting glycose >100 mg/dl, arterial hypertension, and triglycerides >150 mg/dl) and OSA.

**Table 4:** Association between metabolic syndrome components and presence or absence of obstructive sleep apnea

Variables	AHI <5	AHI ≥5		95% CI		
	(*n* = 20)	(*n* = 63)	OR	Lower	Upper	*P* value
HDL <40/50	16 (80)	62 (98)	15.5	1.6	148.4	**
Fasting glycose >100 mg/dl	9 (45)	55 (87)	8.4	2.6	26.5	***
Arterial hypertension	12 (60)	61 (96)	20.3	3.8	107.8	***
Triglycerides >150 mg/dl	10 (50)	62 (98)	62.0	7.1	538.3	***

Number of subjects (% in the group)

*AHI* apnea–hypopnea index, *CI* confidence interval, *OR* odds ratio, *HDL* high-density lipoprotein-cholesterol

** P < 0.01; *** P < 0.001

**Fig. 4:**
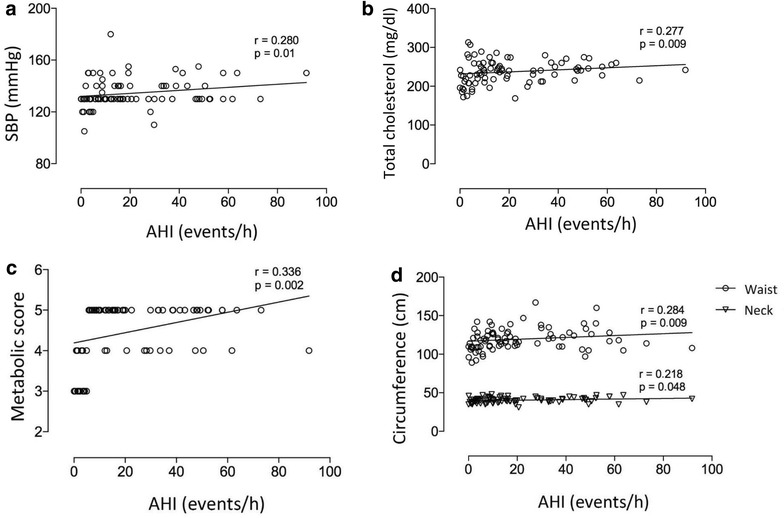
Correlation of apneia/hypopneia index and systolic blood pressure, cholesterol total, metabolic score and circumferences. SBP = systolic blood pressure; AHI = apneia/hypopneia index; mmHg = millimeters of mercury; mg/dl = milligrams per deciliters; cm = centimeters; h = hour. A: correlation of SBP and AHI; B: correlation of total cholesterol and AHI; C: correlation of metabolic score and AHI; D: correlation of circumference and AHI

When the correlation between the variables is analyzed, it is possible to verify that the OSA has significant correlation with metabolic score (r = 0.336; P = 0.002), neck circumference (r = 0.218; P = 0.048), basal SBP (r = 0.280; P = 0.01), TC (r = 0.277; P = 0.011) and abdomen circumference (r = 0.284; P = 0.009), according Fig. 4.

## Discussion

Obesity is one of the major public health problems in developed and developing countries, causing a series of respiratory and metabolic changes. MetS is an association of metabolic and cardiovascular disorders including central obesity, insulin resistance, dyslipidemia and hypertension in the same patient. OSA is a common clinical condition present in patients with MetS. Recent studies have found that more than 60% of patients with MetS also have OSA and that they exhibit similar pathophysiological substrates for CVD, where increased blood pressure is a common consequence [43, 44].

This cross-sectional study characterized clinically a population of severely obese female patients on the waiting list for bariatric surgery and found a high prevalence of Z Syndrome. In addition to routine clinical exams, including blood biochemistry, PSG was also performed in all patients enrolled in the study.

Regarding the demographic and clinical characteristics of the studied population, we can observe that there was no significant difference for the variables of age, weight and BMI, showing the homogeneity of the population even when stratified by groups according to the presence or absence of OSA and according with its gravity. However, it is worth mentioning the significant difference in hip and neck circumference measurements, presenting a considerable increase according to the AHI increase. Regarding the clinical and metabolic variables, a significant difference was observed only for the values of SBP and glycose among patients with and without OSA.

These findings are in agreement with the literature showing a considerable association of MetS with obesity, which are accompanied by increased risk of CVD and type 2 diabetes mellitus, highlighting dyslipidemia, hypertension and elevated glycose levels [7, 45].

For the physiological sleep variables, total sleep time, sleep efficiency and times in the different stages of sleep recorded during PSG, no significant difference was observed, showing that all the patients slept long enough for a good record, presenting an efficiency of normal sleep. However, for most of the variables related to sleep respiratory events, oxyhemoglobin saturation and maximum heart rate, a significant difference was observed. We would like to draw attention to the average saturation of oxyhemoglobin during wakefulness and during sleep and also to the percentage of sleep time below 80 and 70% of oxyhemoglobin saturation.

In the study of Venkateswaran and Shankar conducted in 2006 in a university hospital in Singapore involving 35 patients with MetS observed a prevalence of 62.5% of OSA verified by PSG [46]. In another study, in 2010 Agrawal et al. [47] observed a prevalence of 82% of Z Syndrome in a sample of 272 patients with MetS undergoing PSG. These authors also observed that the prevalence of MetS increases according to the severity of the proven OSA through AHI. In 2013, Barreiro et al. [48] found a prevalence of 82.2% of OSA and a significant association with the components of MetS in a sample of 141 patients with MetS in a hospital in Barcelona.

Our results corroborate these three studies, showing a prevalence of 75.8% of Z Syndrome in patients with MetS and a tendency according to the severity of AHI. A prevalence of 27.7% for mild OSA, 20.4% for moderate OSA, and 27.7% for severe OSA was observed. An association of AHI severity with all components of MetS was also observed, but there was a significant difference only for SBP and for glucose levels.

Current scientific evidence suggests that OSA contributes independently to increased cardiometabolic risks, drawing the attention of healthcare professionals involved in the presence of metabolic dysfunction. During the apneic event hypoxemia occurs associated with hypercapnia, with consequent stimulation of the sympathetic nervous system causing peripheral vasoconstriction. There is also a reduction in intrathoracic pressure and a decrease in pre and post cardiac load.

After restoration of the ventilatory flow, the circulating volume (preload) is increased during vasoconstriction, causing repetitive elevations of blood pressure during the night. Chronically, sustained elevations of blood pressure can be observed due to stimulation of sympathetic autonomic activity, reduction of parasympathetic activity, reduction of nitric oxide, and release of endothelin [49].

Hyperaldosteronism may be a cause of resistant hypertension in patients with OSA. Activation of the renin-angiotensin system, inflammation, insulin resistance, decrease in baroceptor sensitivity, endothelial dysfunction, oxidative stress, and hyperleptinemia may also be implicated in the development of hypertension [31].

Another important finding of this study was the verification of the association between the components of the metabolic syndrome and the presence of OSA. Significant OR values were observed for HDL, fasting glycose, systemic arterial hypertension and hypertriglyceridemia. These data confirm the deleterious impact of OSA on the metabolic and inflammatory markers of the metabolic syndrome, proposed by Drager et al. [43].

In this study, the prevalence of excessive daytime sleepiness (EDS) was verified through the Epworth sleepiness scale [50, 51] and risk for OSA presence through the Berlin clinical questionnaire [52]. The mean values of SED were 10.5 ± 7 demonstrating a value considered normal for its presence. However, a low risk for OSA was observed in only one patient, although the prevalence of OSA was 75.9%.

However, we believe that longitudinal population studies are needed to prove the causal relationship of OSA to metabolic disorders or otherwise, as well as controlled randomized multicenter studies to confirm the beneficial effect of positive airway pressure therapy on metabolic disorders In individuals with OSA.

## Conclusions

According to the study, we can conclude that Z syndrome presents a high prevalence in a female population with MetS and a considerable severity according to the presence of OSA. Therefore, patients with MetS should be investigated for the presence of sleep disturbances and, if observed, referred for specific treatment.

## Abbreviations

OSA: obstructive sleep apnoea
MetS: metabolic syndrome
NCEP ATP III: national cholesterol education program adult treatment panel III
LDL: low-density lipoprotein
CVD: cardiovascular disease
HDL: high-density lipoprotein
BMI: body mass index
AHI: apnea/hypopnea index per hour of sleep
PSG: polysomnography
TC: total cholesterol
EEG: electroencephalogram
EOG: electrooculogram
EMG: electromyogram
EKG: electrocardiogram
AASM: Americam Association of Sleep Medicine
SBP: systolic blood pressure
HR: heart rate
EDS: excessive daytime sleepiness
